# Supplementary material for: Scalable lipid droplet microarray fabrication, validation, and screening
Source: PLoS One. 2024 Jul 5;19(7):e0304736. doi: 10.1371/journal.pone.0304736 (PMC11226032; doi:10.1371/journal.pone.0304736)
Supplement: Attachment — Submitted filename: Response to reviwers.docx [file pone.0304736.s002.docx]

We have addressed all the comments as highlighted in red below.

Journal Requirements:

When submitting your revision, we need you to address these additional requirements.

1. Please ensure that your manuscript meets PLOS ONE's style requirements, including those for file naming. The PLOS ONE style templates can be found at

[https://journals.plos.org/plosone/s/file?id=wjVg/PLOSOne_formatting_sample_main_body.pdf](https://urldefense.com/v3/__https:/journals.plos.org/plosone/s/file?id=wjVg*PLOSOne_formatting_sample_main_body.pdf__;Lw!!PhOWcWs!21n4makGXfePHP83fC2J8J2rnlgCg09D9vELQUaJLEIT114s20ExMd2RlUyavtGEzC_ZprY_8WzpqbnyKQ0$) and

[https://journals.plos.org/plosone/s/file?id=ba62/PLOSOne_formatting_sample_title_authors_affiliations.pdf](https://urldefense.com/v3/__https:/journals.plos.org/plosone/s/file?id=ba62*PLOSOne_formatting_sample_title_authors_affiliations.pdf__;Lw!!PhOWcWs!21n4makGXfePHP83fC2J8J2rnlgCg09D9vELQUaJLEIT114s20ExMd2RlUyavtGEzC_ZprY_8WzpeALJURA$)

We have checked that the revised manuscript meets PLOS ONE’s style requirements, including those for file naming.

2. We noticed you have some minor occurrence of overlapping text with the following previous publication(s), which needs to be addressed:

10.1016/j.nano.2009.04.002

In your revision ensure you cite all your sources (including your own works), and quote or rephrase any duplicated text outside the methods section. Further consideration is dependent on these concerns being addressed

We carefully compared our paper with the referenced publication (10.1016/j.nano.2009.04.002) and found no overlapping text. Perhaps the wrong reference was given? We ran our manuscript through Turnitin and only found overlapping text with the preprint for this manuscript that is available on an archive.

3. We note that the grant information you provided in the ‘Funding Information’ and ‘Financial Disclosure’ sections do not match.

When you resubmit, please ensure that you provide the correct grant numbers for the awards you received for your study in the ‘Funding Information’ section.

We have checked that these two sections match.

4. Please expand the acronym “NIH” (as indicated in your financial disclosure) so that it states the name of your funders in full.

This information should be included in your cover letter; we will change the online submission form on your behalf.

We have expanded the acronym and included it in our coverletter.

5. Thank you for stating the following financial disclosure:

"NIH R01 GM107172"

Please state what role the funders took in the study.  If the funders had no role, please state: "The funders had no role in study design, data collection and analysis, decision to publish, or preparation of the manuscript."

If this statement is not correct you must amend it as needed.

Please include this amended Role of Funder statement in your cover letter; we will change the online submission form on your behalf.

We have included this statement in the coverletter.

6. We noted in your submission details that a portion of your manuscript may have been presented or published elsewhere. "A previous version of this paper has been uploaded to the following preprint archive:

arXiv:2210.07377v1 Available from doi.org/10.48550/arXiv.2210.07377 (Preprint)." Please clarify whether this [conference proceeding or publication] was peer-reviewed and formally published. If this work was previously peer-reviewed and published, in the cover letter please provide the reason that this work does not constitute dual publication and should be included in the current manuscript.

A previous version of this manuscript was uploaded to a preprint archive. It was not peer-reviewed and does not constitute dual publication as it is a preprint archive.

7. When completing the data availability statement of the submission form, you indicated that you will make your data available on acceptance. We strongly recommend all authors decide on a data sharing plan before acceptance, as the process can be lengthy and hold up publication timelines. Please note that, though access restrictions are acceptable now, your entire data will need to be made freely accessible if your manuscript is accepted for publication. This policy applies to all data except where public deposition would breach compliance with the protocol approved by your research ethics board. If you are unable to adhere to our open data policy, please kindly revise your statement to explain your reasoning and we will seek the editor's input on an exemption. Please be assured that, once you have provided your new statement, the assessment of your exemption will not hold up the peer review process.

The relevant data is included in the manuscript.

Additional Editor Comments :

Thank you for submitting your manuscript to PLOS ONE. After careful consideration, we feel that the work is timely, and the manuscript has merit but does not fully meet PLOS ONE’s publication criteria as it currently stands. Therefore, we invite you to submit a revised version of the manuscript that could address the points raised by all the reviewers. The manuscript requires a major revision prior to a final decision.


[Note: HTML markup is below. Please do not edit.]

Reviewers' comments:

Reviewer's Responses to Questions

**Comments to the Author**

1. Is the manuscript technically sound, and do the data support the conclusions?

The manuscript must describe a technically sound piece of scientific research with data that supports the conclusions. Experiments must have been conducted rigorously, with appropriate controls, replication, and sample sizes. The conclusions must be drawn appropriately based on the data presented.

Reviewer #1: Yes

Reviewer #2: Yes

2. Has the statistical analysis been performed appropriately and rigorously?

Reviewer #1: Yes

Reviewer #2: Yes

3. Have the authors made all data underlying the findings in their manuscript fully available?

The [PLOS Data policy](https://urldefense.com/v3/__http:/www.plosone.org/static/policies.action*sharing__;Iw!!PhOWcWs!21n4makGXfePHP83fC2J8J2rnlgCg09D9vELQUaJLEIT114s20ExMd2RlUyavtGEzC_ZprY_8Wzpfw0zdog$) requires authors to make all data underlying the findings described in their manuscript fully available without restriction, with rare exception (please refer to the Data Availability Statement in the manuscript PDF file). The data should be provided as part of the manuscript or its supporting information, or deposited to a public repository. For example, in addition to summary statistics, the data points behind means, medians and variance measures should be available. If there are restrictions on publicly sharing data—e.g. participant privacy or use of data from a third party—those must be specified.

Reviewer #1: Yes

Reviewer #2: Yes

4. Is the manuscript presented in an intelligible fashion and written in standard English?

PLOS ONE does not copyedit accepted manuscripts, so the language in submitted articles must be clear, correct, and unambiguous. Any typographical or grammatical errors should be corrected at revision, so please note any specific errors here.

Reviewer #1: Yes

Reviewer #2: Yes

5. Review Comments to the Author

Please use the space provided to explain your answers to the questions above. You may also include additional comments for the author, including concerns about dual publication, research ethics, or publication ethics. (Please upload your review as an attachment if it exceeds 20,000 characters)

Reviewer #1: In this manuscript, authors have fabricated liquid droplet microarray for cellular validation and screening using nanointaglio. And, the authors have succeeded to screen the screening. From these achievements, this manuscript seems useful for high-throughput screening for cell analysis. However for publication of this manuscript, authors should reconsider the several points.
1. The stability and lifetime of droplets. Is these droplets can be printed on the substrate stably? If possible, the life time also should be discussed.

The following discussion of the stability and lifetime of the droplets has been added to the manuscript

“Phospholipid droplet arrays are stable at low humidity for at least several weeks, but when exposed to high humidity the lipids can spread on the surface.[60] The triglyceride based formulations appear more stable when exposed to humidity although further studies are necessary to determine the extent of their stability for shipping purposes.[38]”

“ Other formulations have previously been shown to be stable in aqueous mediums for over 24 hours.[69] However, there are several time-limiting factors that should be considered when discussing stability and release of encapsulations including the pH and the temperature of the aqueous environment.[69, 70] “

2. Are the cell density affect to the screening efficiency?

The following discussion of the cell density has been added to the manuscript:

“Variation in cellular uptake is likely to depend on variation in cellular contact with the arrays, as well as heterogeneities within the cell population, such as differential stages of the cell cycle and gene expression. Furthermore, discrepancies in cell density can influence several different processes that could be considered vital for drug screening, [73] with studies showing that cell plates of differing levels of confluence, show varied amounts of expression for specific proteins.[74] Depending on whether these proteins, serve cellular functions related to metabolism or are related to pathways associated with the drug itself, there may be effects on the efficacy of the drug screen. The cell density also determines how many cells will be present on an individual spot. Depending on the variance and effect size, a sample size of approximately 50 cells per spot appears sufficient for two sample comparisons with the assays presented here.[75]”

3. If possible, after the printing with different pressures, differences of sizes of droplet should be indicated using SEM imaging.

We carried out SEM and added the following figure and caption:

“

**Fig 3. Micrographs of hexanoic acid / castor oil (hex/cas) droplet arrays prepared in the optimal pressure range.** (a) Representative fluorescence image (b) and (c) are scanning electron microscopy (SEM) images of arrays with a magnified view of the specific location boxed in white of hexanoic acid / castor oil (hex/cas) droplets stamped using vertical pressure of approximately 30 N/cm^2^. Oils were doped with rhodamine-PE for visualization.”

We were not able to distinguish the different sizes in the SEM due to the low aspect ratios of the drops. However, we have previously published detailed AFM studies on the relation between droplet size and fluorescence intensities, and have added the following text to the manuscript referencing that:

“ We have previously shown the material integration capabilities by printing spots with two different fluorophores.[45] We have also demonstrated a linear the relationship between the multilayer thickness or heights of our subcellular nanostructures measured by atomic force microscopy (AFM) and the intensity of fluorescence emission they produce.[46] This calibration has allowed us to determine droplet volumes and therefore quantify dosage in terms of fluorescence intensity.[44] ”

Reviewer #2: The work reported in the manuscript is interesting in terms of both the method to prepare droplets, and the cell responses to droplets. The former is the focus of the manuscript. In forming the droplets, the first step is quite ordinary, but it is clever to split the ink droplets by using intaglio stamp. I have the following comments.

1. Figure 2 may better show the images of the droplet array prepared under the optimal pressure of 16 N/m^2, along with current representative images. It is important to show 100% uniformity droplets.

Figure 2c shows an example of arrays printed with the optimal pressure. We have also added a new figure 3 showing fluorescence and SEM images of uniform droplets. We have clarified the definition of uniformity as follows:

“we define “uniformity” as the percentage of patterned area covered with ink from the stamp that has transferred individual subcellular nanostructures(~5µm) each to the surface, with dosage being quantified after printing”

“Printing pressure of 16-32 N/cm2 and stamp times between 130 and 200 seconds were found to adhere with optimal uniformity > 95% “

“**Figure 2.** f) A bar graph showing the vertical pressure of 16 to 32 N/cm2 had the highest average uniformity percentage over the span of 130-200 seconds. “

2. What is the interfacial tension between aqueous medium and the lipid droplets? This tension determines whether the deformed droplets can readily resume a spherical cap shape.

The following discussion of the lipid droplet shape in terms of interfacial tension has been added:

“When deformed droplets experience this immersion, they attempt to form a spherical cap shape in an effort to reduce interfacial tension.[67] The role this phenomenon plays is directly related to the stability of the emulsion itself. The interfacial energy present at the interface is determined by multiple factors including the presence and viscosity of a surfactant and the temperature of the droplets. [38, 68] Previous studies have shown that using surfactant co-emulsifiers can help improve the stability of the droplets during immersion by lowering the amount of interfacial energy present in the system. [38] The use of castor oil/hexanoic acid in our formulations is to provide further stability to our droplet formulation. ”

3. The authors pointed out that the droplets are not stable as they are immersed into the aqueous medium. The reason for such instability is the development of four phase contact line, which is unavoidable in the process of bringing oil-like droplets into water, or vice verse. See the detailed mechanism: Splitting droplets through coalescence of two different three-phase contact lines. [Soft matter 15 (30), 6055-6061 (2019)](https://pubs.rsc.org/en/content/articlelanding/2019/sm/c9sm00638a). The approach taken by the authors to improve the stability of the droplets is ok. An alternative way may be to improve the viscosity of the droplets, or control the speed for immersion. The authors may provide these possibilities in their outlook for future work.

We thank the reviewer for this insight and relevant reference (now our reference 61), which we have added to the manuscript as follows:

“ When organic droplets on a surface are immersed into solution, they are inevitably exposed to an air water interface, which can disrupt the droplets through dissolution and exposure to mechanical forces.[60, 61] Fluid lipid multilayer patterns on surfaces can therefore be destroyed upon immersion into aqueous solution unless care is taken to prevent this.[39, 43, 45, 47, 63] …Faster immersion results in less exposure to the interface, and higher ink viscosity can be expected to stabilize the droplets upon immersion.[61] Previous works have improved immersion stability through freezing the droplets [45, 47, 66] and identification of presumably more viscous and less amphiphilic triglyceride-based formulations can allow more stable immersion.[33, 38, 39, 44, 47, 64, 65] ”

4. The surface gettability of the stamps would play an important role in precisely distributing the droplet liquid. What are the advancing and receding contact angles of the stamp materials?

We measured the contact angles on the stamp and substrates and included them as follows in the mansucript:

“We measured the advancing and receding contact angles on flat PDMS surfaces to be 82 +/- 4° and 77 +/- 4°, respectively, and advancing and receding contact angles measured on the glass substrates were 61° +/- 4° and 42° +/- 2°, respectively. “

6. PLOS authors have the option to publish the peer review history of their article ([what does this mean?](https://urldefense.com/v3/__https:/journals.plos.org/plosone/s/editorial-and-peer-review-process*loc-peer-review-history__;Iw!!PhOWcWs!21n4makGXfePHP83fC2J8J2rnlgCg09D9vELQUaJLEIT114s20ExMd2RlUyavtGEzC_ZprY_8WzpETbSDDs$)). If published, this will include your full peer review and any attached files.


If you choose “no”, your identity will remain anonymous but your review may still be made public.


**Do you want your identity to be public for this peer review?** For information about this choice, including consent withdrawal, please see our [Privacy Policy](https://urldefense.com/v3/__https:/www.plos.org/privacy-policy__;!!PhOWcWs!21n4makGXfePHP83fC2J8J2rnlgCg09D9vELQUaJLEIT114s20ExMd2RlUyavtGEzC_ZprY_8WzpdCAMVhE$).

Reviewer #1: No

Reviewer #2: **Yes:** Xuehua Zhang

[NOTE: If reviewer comments were submitted as an attachment file, they will be attached to this email and accessible via the submission site. Please log into your account, locate the manuscript record, and check for the action link "View Attachments". If this link does not appear, there are no attachment files.]

While revising your submission, please upload your figure files to the Preflight Analysis and Conversion Engine (PACE) digital diagnostic tool, [https://pacev2.apexcovantage.com/](https://urldefense.com/v3/__https:/pacev2.apexcovantage.com/__;!!PhOWcWs!21n4makGXfePHP83fC2J8J2rnlgCg09D9vELQUaJLEIT114s20ExMd2RlUyavtGEzC_ZprY_8WzpVgsmbaM$). PACE helps ensure that figures meet PLOS requirements. To use PACE, you must first register as a user. Registration is free. Then, login and navigate to the UPLOAD tab, where you will find detailed instructions on how to use the tool. If you encounter any issues or have any questions when using PACE, please email PLOS at [figures@plos.org](mailto:figures@plos.org). Please note that Supporting Information files do not need this step.

*In compliance with data protection regulations, you may request that we remove your personal registration details at any time.* [*(Remove my information/details)*](https://urldefense.com/v3/__https:/www.editorialmanager.com/pone/login.asp?a=r__;!!PhOWcWs!21n4makGXfePHP83fC2J8J2rnlgCg09D9vELQUaJLEIT114s20ExMd2RlUyavtGEzC_ZprY_8WzpS9JDrEo$)*. Please contact the publication office if you have any questions.*
